# Abnormal L5-S1 Facet Joint Orientation as a Harbinger of Degenerative Spondylolisthesis: A Case Report

**DOI:** 10.7759/cureus.40569

**Published:** 2023-06-17

**Authors:** Collin M Labak, Rohit Mauria, Eric Z Herring, Michael D Shost, Manish K Kasliwal

**Affiliations:** 1 Neurological Surgery, University Hospitals Cleveland Medical Center, Cleveland, USA

**Keywords:** injury biomechanics, surgery, orthopedic surgery, neurosurgery, lumbar spine, transforaminal lumbar interbody fusion, spondylolisthesis, spine surgery

## Abstract

Degenerative spondylolisthesis is a common cause of low back pain and resultant disability in the adult population. The causes of degenerative spondylolisthesis are not entirely understood, though a combination of anatomic and lifestyle factors likely contributes to the development of this pathology. Here, we report a case of a 38-year-old female presenting with low back pain and right lower extremity radiculopathy, found to have degenerative L5-S1 spondylolisthesis, which we postulate developed in part due to the sagittal orientation of her L5-S1 facet joints bilaterally.

## Introduction

Degenerative lumbar spondylolisthesis (DLS) is an extremely common spine condition with incidence estimates ranging from 12% to 19.9% [1]. In this pathology, one vertebral body translates relative to its adjacent counterpart with the potential to cause significant low back pain, neurogenic claudication and/or radiculopathy secondary to instability as well as central canal, lateral recess, or foraminal stenosis [2,3]. Neurogenic claudication results in a constellation of symptoms including cramping back, buttock, and leg pain due to the compression of multiple lumbar nerve roots in the cauda equina, while radiculopathy relates to buttock and leg pain attributable to a single compressed nerve root in the lateral recess or neural foramen [4]. Symptomatic DLS can lead to diminished health-related quality of life (HRQOL) and disability in the adult population [5,6]. Following failure of an appropriate trial of non-operative treatment, surgical treatment consists of either decompression alone or decompression with instrumented fusion through an open or minimally invasive approach depending on various pathological and patient-related factors [7-10]. DLS is most common at the L4-5 level followed by the L5-S1 level [2,4]. However, isthmic spondylolisthesis secondary to pars interarticularis defect accounts for the majority of cases of spondylolisthesis at L5-S1, rather than DLS [11]. Here, we present the case of a young female with mobile degenerative L5-S1 spondylolisthesis secondary to the presence of abnormal facet joint morphology/anatomy as a predisposing factor for the early development of this DLS.

## Case presentation

**Figure 1:**
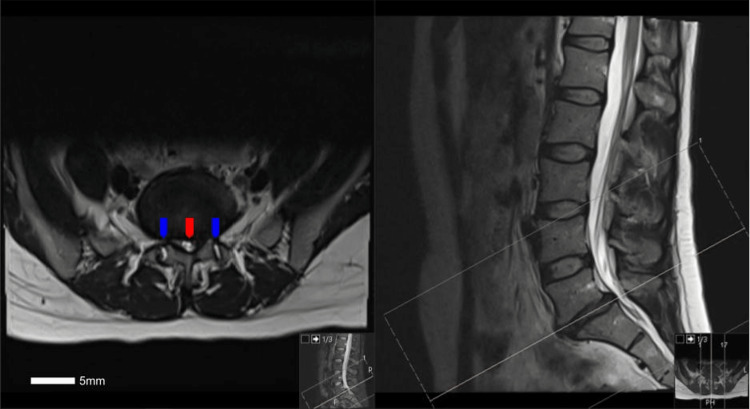
Axial (left) and sagittal (right) T2-weighted MRI of the lumbar spine without contrast at the level of L5-S1, demonstrating severe central canal and lateral recess stenosis (red arrow) with bilateral foraminal stenosis, as well as facet joint effusions (blue arrows)

The patient was a 38-year-old female without a significant medical history who presented to our clinic in the setting of low back pain, neurogenic claudication and right lower extremity pain for a duration of 18 months. She tried medications including membrane stabilizers, muscle relaxants, and nonsteroidal anti-inflammatory drugs with little relief, and had attempted physical therapy as well as epidural steroid injections with only temporary relief. On physical examination, the patient did have full strength throughout her bilateral lower extremities; however, a sensory examination demonstrated altered sensation involving the right lower extremity along the L5 and S1 dermatomes. Magnetic resonance imaging (MRI) of the lumbar spine demonstrated significant disc degeneration and collapse at the L5-S1 level along with disc herniation resulting in moderate to severe L5-S1 central canal and foraminal stenosis along with presence of grade 1 L5-S1 spondylolisthesis (Figure 1).

**Figure 2:**
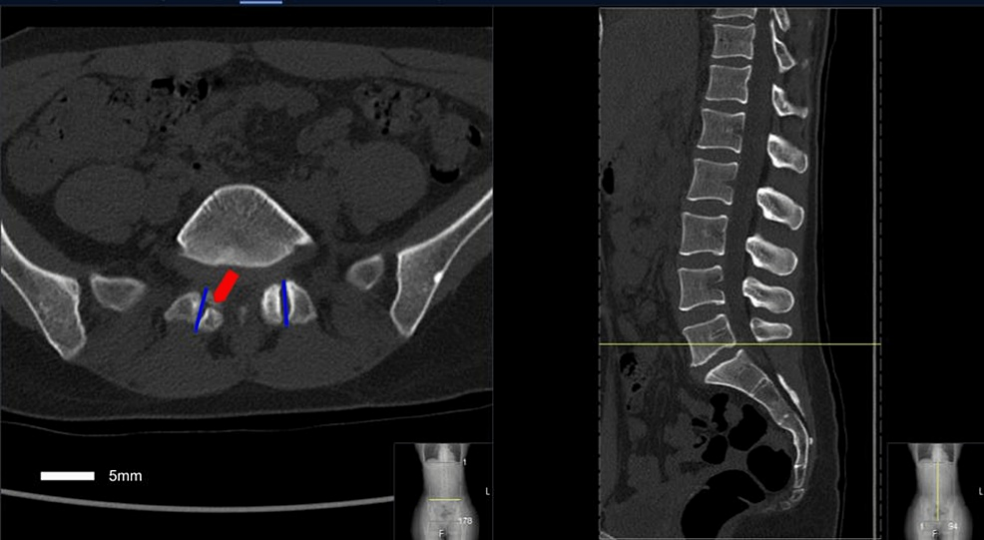
An axial (left) and sagittal (right) CT scan of the lumbar spine at the L5-S1 level Note the pars defect on the right (red arrow), as well as the sagittal orientation of the L5-S1 facet joint line (blue lines).

Computed tomography (CT) of the lumbar spine was performed to understand further anatomical details and rule out pars defect that demonstrated the presence of abnormal/atypical facet joint anatomy with the more sagittally oriented L5-S1 facet joint (Figure 2).

The facet joint at L5-S1 demonstrated an angle relative to the midsagittal axis of 1.6°, which is substantially more sagittal than what would be expected of a L5-S1 joint that is typically coronally oriented (Table 1).

**Table 1:** Facet tropism of the lumbar spine Corresponding angles of each lumbar facet joint measured on the patient’s CT relative to a midsagittal line, or the patient’s facet tropism; the expected progression would include a continued increase in the angle with caudal progression down the lumbar spine.

Level	Angle of the facet joint line relative to the midsagittal line
L1-L2	20.4
L2-L3	24.5
L3-L4	33.4
L4-L5	35.3
L5-S1	1.6

**Figure 3:**
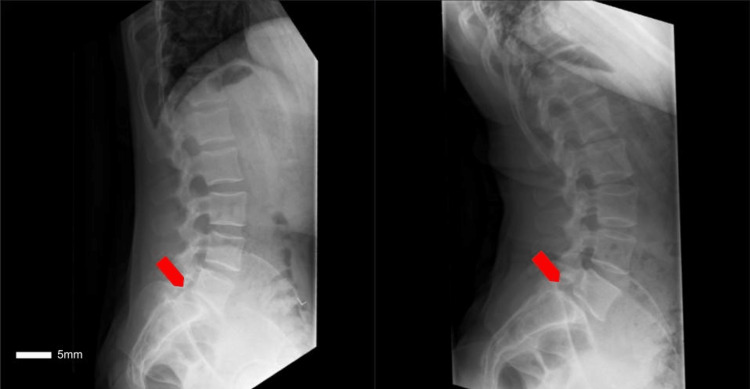
A flexion (left) and extension (right) lateral plain film radiograph of the lumbar spine demonstrating a mobile spondylolisthesis at L5-S1 (red arrows)

Additionally, flexion/extension plain films were obtained that showed a mobile Meyerding Grade I spondylolisthesis at L5-S1 secondary to the inability of the facet joint to prevent translational motion due to the sagittal orientation (Figure 3).

**Figure 4:**
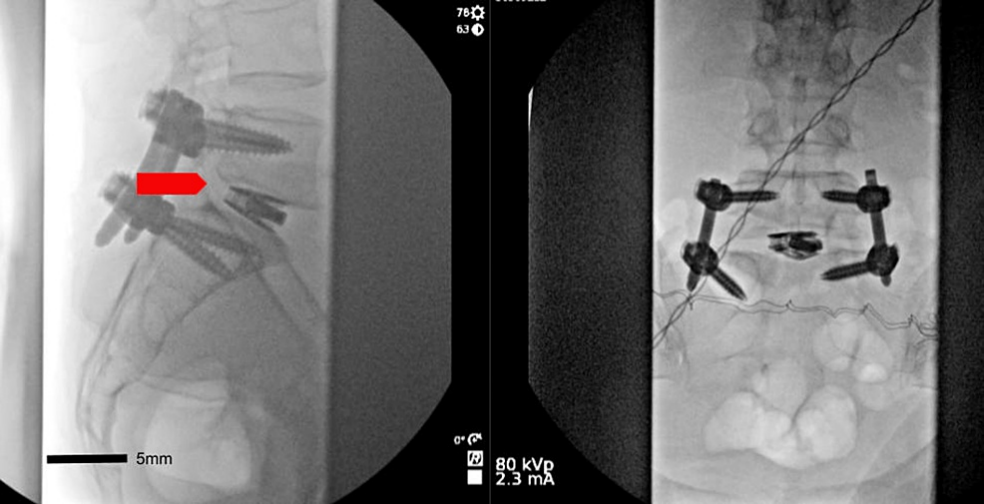
A lateral (left) and anterior/posterior (right) lateral plain film radiograph of the lumbar spine after the placement of the L5-S1 interbody graft and percutaneous pedicle screw fixation, with reduction of the spondylolisthesis (red arrow) seen on the lateral film

After a discussion of risks and benefits of surgical intervention, the patient ultimately underwent an L5-S1 minimally invasive decompression and transforaminal lumbar interbody fusion (TLIF) with pedicle screw fixation using intraoperative 3D navigation. The surgery was completed without complications with complete reduction of the spondylolisthesis (Figure 4). The patient was discharged with significant improvement in her radiculopathy.

## Discussion

This case demonstrates an atypical characteristic of degenerative spondylolisthesis. Our patient did not meet the typical demographics of degenerative spondylolisthesis, as DLS presents most commonly at the L4-L5 level in patients older than 50 years of age [12]. Also, she lacked the typical pars defect that accounts for the majority of cases of L5-S1 isthmic spondylolisthesis in younger populations [13]. Therefore, it is important to assess anatomic factors that may have contributed to the premature development of this pathology.

Facet tropism, the variation in facet joint orientation in the sagittal plane, has been sparsely studied and is often overlooked in the development of lumbar degenerative pathology [14]. Facet tropism in our patient demonstrated a general increase in angulation until an abruptly sagittal facet joint at the L5-S1 level (Table 1). Previous research has primarily explored facet orientation and tropism in relation to lumbar disc herniation among young adults [15]. While there is limited evidence suggesting that facet tropism alone does not contribute to lumbar stenosis, it has been proposed as an independent predictor of progressive DLS by Yoshihara [2,16]. Multiple studies have demonstrated an association between an increased sagittal facet joint angulation and an elevated risk of developing spondylolisthesis [17,18]. Specifically, studies by Samartzis et al. and Guo et al. found that facet angulations exceeding 58 and 60 degrees, respectively, were indicative of a greater likelihood of spondylolisthesis development. Furthermore, Connolly et al. in a study of adolescent tennis players found that larger facet joint angles were associated with higher rates of spondylolysis that might predispose them to future pars abnormalities [19].

Leng and colleagues conducted a study examining the relationship between spinopelvic type and facet joint orientation in the development of lumbar degenerative spondylolisthesis. They discovered that the facet joint morphology undergoes dynamic changes, with remodeling of facet joint orientation accompanying the progression of spondylolisthesis [20]. Facet joint morphology and angulation should be considered when conservatively managing symptomatic patients with degenerative pathologies of the lumbar spine in order to identify those who may be predisposed to further progression of their degenerative disease process.

## Conclusions

We believe that the presence of abnormally sagittally oriented facet joints, as described in this case, at the L5-S1 level leads to the development of L5-S1 degenerative lumbar spondylolisthesis, even in the absence of florid pars defects. Considering facet joint angulation may improve patient counseling and treatment selection, as a sagittal facet joint orientation can potentially accelerate degenerative spondylolisthesis. Moreover, the atypical joint anatomy can pose challenges in accurately identifying intraoperative landmarks, particularly during minimally invasive techniques that offer limited visualization. Therefore, obtaining preoperative CT scans and intraoperative navigation are increasingly important in young patients with spondylolisthesis. This case serves as an important teaching point for a thorough assessment of anatomic nuances that affect patient management.
